# A 40-MHz Ultrasound Transducer with an Angled Aperture for Guiding Percutaneous Revascularization of Chronic Total Occlusion: A Feasibility Study

**DOI:** 10.3390/s18114079

**Published:** 2018-11-21

**Authors:** Junsu Lee, Jin Ho Chang

**Affiliations:** 1 Department of Electronic Engineering, Sogang University, Seoul 04107, Korea; leejs@sogang.ac.kr; 2 Department of Biomedical Engineering, Sogang University, Seoul 04107, Korea

**Keywords:** chronic total occlusion, atherosclerosis, percutaneous coronary intervention (PCI), guidewire crossing, intravascular ultrasound (IVUS), forward-looking IVUS, high-frequency ultrasound, ultrasound transducers

## Abstract

Complete blockage of a coronary artery, called chronic total occlusion (CTO), frequently occurs due to atherosclerosis. To reopen the obstructed blood vessels with a stent, guidewire crossing is performed with the help of angiography that can provide the location of CTO lesions and the image of guidewire tip. Since angiography is incapable of imaging inside a CTO lesion, the surgeons are blind during guidewire crossing. For this reason, the success rate of guidewire crossing relies upon the proficiency of the surgeon, which is considerably reduced from 69.0% to 32.5% if extensive calcification, not penetrated by a guidewire, exists in CTO lesions. In this paper, a recently developed 40-MHz forward-looking intravascular ultrasound (FL–IVUS) transducer to visualize calcification within CTO lesions is reported. This transducer consists of a single element angled aperture and a guidewire passage. The aperture is spherically deformed to have a focal length of 3 mm in order to improve spatial resolution of FL–IVUS images. The angle between the beam direction and the axis of rotation is designed to be 30° to effectively visualize calcification within a CTO lesion as well as the blood vessel wall. The experimental results demonstrated that the developed FL–IVUS transducer facilitates visualization of calcification within CTO lesions and makes it possible to help the surgeon make decisions about whether to push the guidewire in order to cross the lesion or to change the surgical procedure.

## 1. Introduction

Atherosclerosis, a buildup of plaque in artery walls, develops gradually, thus causing arteries to narrow [1]. Chronic total occlusion (CTO), defined as complete blockage of a coronary artery, frequently occurs due to atherosclerosis; CTO was found in 33–50% of patients diagnosed with severe coronary artery disease in angiography [2]. Before the obstruction is complete, side-looking intravascular ultrasound (SL–IVUS) in conjunction with angiography can be used to measure the degree of stenosis, assess the possibility of vulnerable plaque, and guide stent implantation [3]. Recently, new SL–IVUS transducers and imaging algorithms have been developed to further enhance its usefulness in clinics [4,5,6,7]. A dual-element SL–IVUS transducer for ultrasound elastrography [8] and an SL–IVUS transducer integrated with optical fiber for photoacoustic imaging [9] have been developed to identify plaque compositions. For CTO lesion visualization, in contrast, angiography is the only diagnostic imaging tool commonly used in clinics. Since angiography can provide the location of CTO lesions and the image of guidewire tip, this imaging modality is useful for identifying the entry point of a CTO lesion during guidewire crossing that is an essential step in reopening the obstructed blood vessels with a stent [2,10]. However, continuously exposing the surgeon to radiation is burdensome because angiography should be used consistently during the surgery to locate a guidewire. Additionally, the success rate of guidewire crossing relies upon the proficiency of the surgeon because no visual information inside a CTO lesion is available in angiograms. For example, the success rate is considerably reduced from 69.0% to 32.5% when CTO lesions contain extensive calcification that is not penetrated by a guidewire [10,11]. This is mostly attributed to the fact that angiography is unable to identify calcified lesions. If real-time visualization of calcification within CTO lesions is possible, the surgeon can avoid the area when pushing the guidewire to cross the lesions or change the surgical procedure in an alternative approach, such as retrograde guidewire crossing in which collateral channels are used to allow a guidewire to reach the distal entry of a CTO lesion [12,13]. Note that retrograde technology provides performance similar to the conventional antegrade approach at the expense of long surgical time and more radiation exposure [14].

Although SL–IVUS imaging has been used to identify guidewire position within a coronary artery and distinguish false lumens from true lumens before guidewire crossing [15], forward-looking intravascular ultrasound (FL–IVUS) imaging is a suitable tool for locating the exact entry point of a CTO lesion before a guidewire reaches this point and for visualizing inside the CTO lesion. The first FL–IVUS transducer was developed for forward-viewing sector-scanning imaging [16]; a flat aperture was mechanically wobbled to obtain front view images, and the size of the transducer was relatively large, i.e., 4 mm in diameter, due to its mechanical module. To solve the problems of the large size and mechanical scanning, a FL–IVUS ring-annular array was proposed [17] and developed [18], but high fabrication costs and imaging system complexity may degrade its clinical usefulness because IVUS transducers have to be disposable. On the other hands, forward-looking Capacitive Micromachined Ultrasonic Transducer (CMUT) ring arrays were proposed despite high manufacturing costs due to the complexity of sensor structure and imaging modules [19,20]. In addition, the high Direct Current (DC) bias voltage required for operating CMUTs is a risk factor, especially when the sensor should be inserted inside the body. The insulation from the DC bias also has a negative effect on the performance of CMUTs [21]. Recently, a steerable FL–IVUS catheter containing a single-element ultrasound transducer has been developed; 16 wires are used to steer the transducer and an optical shape sensing system measures the current position of the transducer for image reconstruction [22]. This catheter may be clinically useful because it is able to construct C-scan and 3D ultrasound images of CTO lesions. However, fast scanning time should be guaranteed, and it should be verified that including a guidewire in the catheter does not affect steering performance and the increase in catheter size due to a guidewire passage is not a problem in clinical use if the catheter is used for guidewire crossing guidance.

For these reasons, high-frequency single element angled transducers are a good alternative [23] because rotating the transducer can form a conical imaging plane along the vertical direction of the blood vessel [24]. Based on this fact, a few angled transducers were proposed for FL–IVUS imaging [25,26]; the angle between the beam direction and the axis of rotation is larger than 45°. These FL–IVUS transducers are capable of providing the information about the location of the vessel wall inside CTO lesions, but it is not appropriate to obtain information on the presence or absence of calcification within a CTO lesion and, if present, on its location in the CTO lesion. This is so because the semi-vertical angle of a conical imaging plane is too large to accurately predict the trajectory of a guidewire within a CTO lesion in the acquired FL–IVUS images. Furthermore, no studies have been conducted to confirm the possibility of visualizing the calcified region by the FL–IVUS transducers.

Here, we report a recently developed 40-MHz FL–IVUS transducer used for image-guided guidewire crossing. This transducer consists of a single element angled aperture and a guidewire passage. The aperture is spherically deformed to have a focal length of 3 mm in order to improve spatial resolution of FL–IVUS images. The angle between the beam direction and the axis of rotation is designed to be 30° to effectively visualize calcification within a CTO lesion as well as the blood vessel wall. In addition, the relatively small angle enables the surgeons to determine whether calcification is in the pathway of guidewire crossing or not. Using custom blood-vessel-mimicking phantoms constructed for this study, it is demonstrated that the calcification inside the phantom appears in the FL–IVUS images acquired by the developed transducer. The FL–IVUS imaging with the developed transducer is conducted in different situations depending on calcification location in the guidewire pathways. From the experimental results, it is shown that the developed FL–IVUS transducer facilitates visualization of calcification within CTO lesions and makes it possible to help the surgeon make decisions about whether to push the guidewire in order to cross the lesion or to change the surgical procedure.

## 2. Materials and Methods

### 2.1. Concept of the Proposed FL–IVUS Transducer

**Figure 1:**
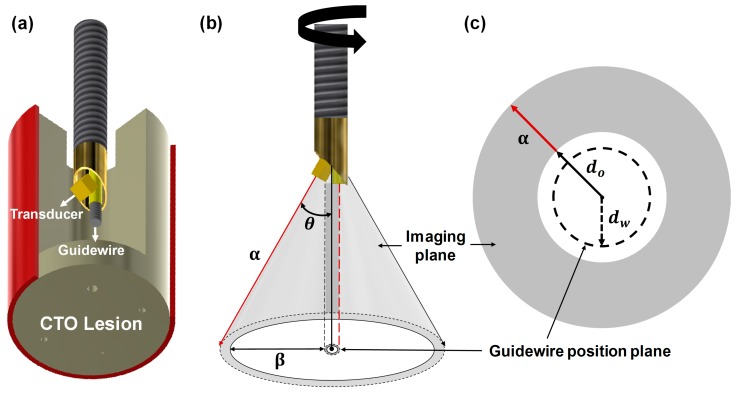
Conceptual illustration of (**a**) the proposed forward-looking intravascular ultrasound (FL–IVUS) transducer consisting of a single-element angled aperture and a guidewire passage, (**b**) the conical imaging plane along the vertical direction of the blood vessel, and (**c**) the FL–IVUS image expected to be displayed on a monitor. *α* and *θ* indicate the slant height and semi-vertical angle of the conical imaging plane. *β* is the closest distance between the imaging plane and the guidewire position plane. *d*_0_ and *d_W_* are the distances from the vertex of the cone to the aperture surface and to the center of the guidewire passage, respectively.

The proposed FL–IVUS transducer has a beveled aperture and a guidewire passage as shown in Figure 1a. When a θ-angled aperture is rotated, a conical imaging plane with a semi-vertical angle θ is formed in the axial direction (see Figure 1b). In this case, the guidewire passage is also rotated, thus forming a cylindrical guidewire position plane. The location of a guidewire in the position plane is determined by a rotation angle. However, the guidewire is always placed on the opposite side of the scanline being acquired by the transducer as shown in Figure 1b; for example, the guidewire will be in the position plane indicated by the red dashed line when the scanline indicated by the red solid arrow is being acquired. Therefore, the path of guidewire crossing does not appear in the FL–IVUS image that will have a similar shape to a SL–IVUS image when it is displayed on a monitor. The difference is that the dead zone in the FL–IVUS image, indicated by a white circle in Figure 1c, is larger than that in a SL–IVUS image because there are no echo signals between the vertex of the cone and the aperture surface (i.e., *d*_0_). In addition, the guidewire position plane is located inside the dead zone (see the dashed circle in Figure 1c). However, when a CTO lesion is close to the transducer, the surgeons can predict from the FL–IVUS image whether calcification is in the pathway of guidewire crossing. Since *d*_0_ is the distance that the aperture surface is apart from the vertex of the cone, the closest distance between the imaging plane and the guidewire position plane can be expressed as

(1) $$\beta =\left(\alpha +d_{0}\right)\sin\left(\theta \right)-d_{W}$$

where *α* and *θ* are the slant height and the semi-vertical angle of the conical imaging plane, and *d_W_* is the radius of the guidewire position plane. In Equation (1), all parameters are determined at the design stage except *α* that is a function of imaging depth. *d_W_* is approximately equal to the radius of a guidewire passage if a rotation axis is the boundary between a guidewire passage and an acoustic stack, and *d*_0_ is determined by the thickness of an acoustic stack *d_AS_*, aperture size *γ*, and *θ*, i.e.,

(2) $$d_{0}=d_{AS}+\frac{\gamma }{2\tan\left(\theta \right)}$$

For example, if *d_AS_* is 0.5 mm, *γ* is 0.5 mm, and *θ* is 30°, *d*_0_ is calculated to be 0.933 mm. In the case of a passage radius of 0.225 mm, from Equation (1), *β* is 0.242 mm when *α* is zero. This value increases by 0.25 mm for every 0.5 mm increase in *α*. Calcification around the guidewire pathway is expected to appear in the FL–IVUS image if the slant height *α* is less than 1 mm. This will be demonstrated below. Note that all of the above values were determined at the design stage of the proposed FL–IVUS transducer.

### 2.2. Design and Fabrication of the FL–IVUS Transducer

**Figure 2:**
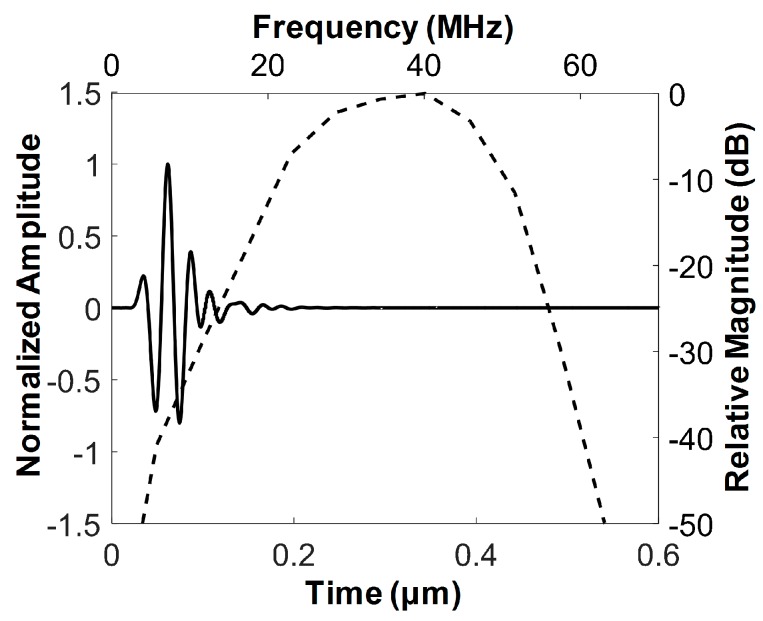
Pulse-echo response (solid line) and its frequency spectrum (dashed line) obtained by simulation using a PiezoCAD program (Sonic Concept, Woodinville, WA, USA).

The acoustic stack was designed to have a backing block, PZT-5H (3203HD, CTS Technology, Sparta, IL, USA) as an active material, and two matching layers. PZT-5H was selected because this material not only has a good electromechanical coupling coefficient, but also is affordable for disposable IVUS transducers and is relatively easy to be spherically shaped for a geometric focus. The element size was chosen to be 0.5 × 0.5 mm^2^, and geometric focus at 3 mm was selected considering a natural focal depth of 2.2 mm at 40 MHz, i.e., 0.339 × (side length of the aperture)^2^/wavelength = 0.339 × (0.5 mm)^2^/38.5 μm [7]. Since the diameter of coronary arteries ranges from 2.0 to 4.5 mm [27], the diameter of a FL–IVUS transducer including a guidewire passage should be much smaller 2 mm. In addition, the element size of a FL–IVUS transducer should be as large as possible to achieve high spatial resolution. To this end, we decided the diameter of the FL–IVUS transducer to be 1 mm, considering the diameter of the guidewire passage, i.e., 0.45 mm. In addition, *θ* was determined to be 30° because this was the smallest angle to integrate the element with a size of 0.5 × 0.5 mm^2^ and the guidewire passage into a 1-mm diameter housing. Note that the smallest possible *θ* in Equation (1) is desirable for FL–IVUS imaging. Conductive epoxy (E-solder 3022, Von Roll USC Inc., Schenectady, NY, USA) was chosen for the backing block to facilitate the connection between the PZT-5H in the acoustic stack and a signal wire. In the case of PZT-5H, theoretical acoustic impedances of two matching layers were calculated to be 9.32 and 2.37 MRayls [7,28]. Among the materials available in our laboratory, the mixture of 2–3 μm silver particles (Sigma-Aldrich Co., Milwaukee, WI, USA) with Insulcast 501 and Insulcure 9 (American Safety Technologies, Roseland, NJ, USA), called silver epoxy in this paper, was chosen for the first matching layer because its acoustic impedance, i.e., 7.334 MRayls, was most similar to the theoretical value. Parylene C (PDS2010, Specially Coating Systems Inc., Indianapolis, IN, USA) of which acoustic impedance is 2.59 MRayls served as the second matching layer. The acoustic properties and thicknesses of those materials are summarized in Table 1. Note that a PiezoCAD software package (Sonic Concept, Woodinville, WA, USA) was used to determine the thickness of each layer. Figure 2 shows the expected pulse-echo response and frequency spectrum of the designed acoustic stack, which were obtained by the PiezoCAD simulation; the center frequency was 36.1 MHz, and the −6 dB fractional bandwidth was 68%. The peak spectral magnitude occurred at 40 MHz.

**Figure 3:**
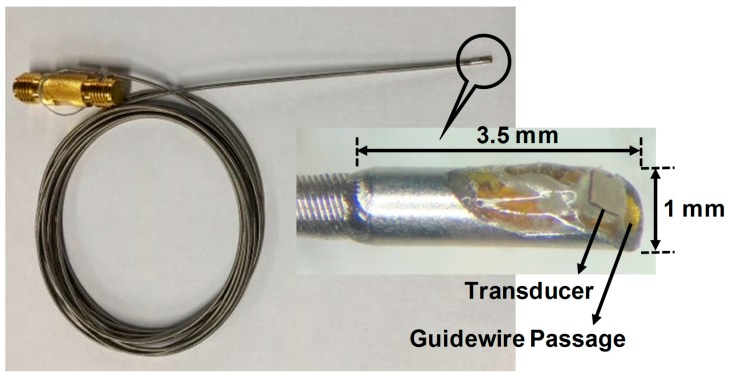
Photographs of the finished FL–IVUS transducer and the zoomed-in version of the white solid circle.

The acoustic stack was firstly fabricated before constructing the FL–IVUS transducer. For this, a bulk PZT-5H of 1.0 × 1.0 cm^2^ was lapped to a desired thickness of 59 μm and sputtered with Cr/Au (500 Å/2000 Å) that acts as an electrode. The silver epoxy was prepared and poured onto the sputtered surface of the PZT-5H surrounded by plastic bars that served as dams. A detailed manufacturing process for the silver epoxy can be found in [29]. The silver epoxy on the PZT-5H was centrifuged at 3000 RPM for 10 min to degas and cured at room temperature for 24 h in a dry box. The desired thickness of the silver epoxy, i.e., 13 μm was achieved by lapping. The backing material, i.e., conductive epoxy, was cast onto the opposite side of the PZT-5H face with the matching layer after constructing the dams. The backing block was cured at room temperature for 24 h and lapped to a target thickness of 414 μm after centrifugation at 3000 RPM for 10 min. Finally, the bulk acoustic stack was diced to a size of 0.5 × 0.5 mm^2^ using a dicing machine (DAD 322, Disco Corp., Tokyo, Japan). An Room-Temperature-Vulcanizing (RTV) molder with a curvature of 3 mm radius was prepared for geometric focus. A piece of the final acoustic stack was placed on the RTV molder, and press-focusing was subsequently perfomed using a steel ball with a radius of 3 mm. The detailed procedure for the press-focusing can be found in [30]. To create a guidewire passage, a polyimide tube with an inner diameter of 410 μm and an outer diameter of 450 μm was used. Since a standard guidewire has a diameter of 360 μm (or 0.014 inch), the polyimide tube can accommodate the movement of standard guidewires. An edge of the backing block was attached to the side of the polyimide tube by using 5 min Epoxy (ITW Polymers Adhesives North America, Danvers, MA, USA) after the angle between the beam direction and the polyimide tube was adjusted to 30°. The attached acoustic stack and polyimide tube were fixed with 5 min Epoxy at the end of a housing tube. As the second matching layer, Parylene C was deposited to the acoustic stack. Note that the thickness of the acoustic stack was 0.5 mm and the size of the finished FL–IVUS transducer was 2.3 mm in diameter. Figure 3 shows the photographs of the finished FL–IVUS transducer and the zoomed-in version of its tip.

## 3. Results and Discussion

### 3.1. Characteristics of the Developed FL–IVUS Transducer

**Figure 4:**
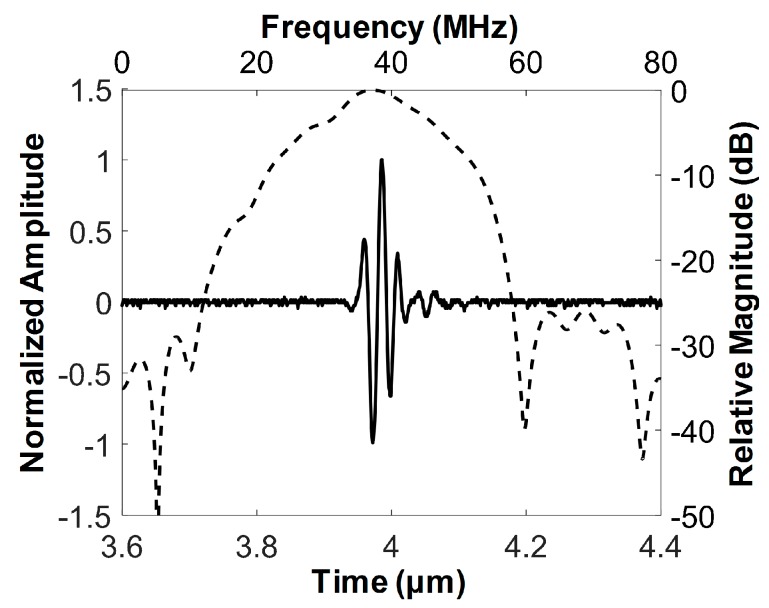
Measured pulse–echo response (solid line) and frequency spectrum (dashed line) of the developed FL–IVUS transducer.

The pulse–echo response was measured to determine the center frequency and −6 dB fractional bandwidth of the developed FL–IVUS transducer. For this, the developed transducer and a polished steel target plate were immersed into a deionized-water-filled container. The target plate was tilted by 30° with respect to the horizontal axis so that the target surface could be perpendicular to the beam direction. The transducer was mounted on an arm connected to a motorized stage (SGSP26-100, SIGMAKOKI Co. Ltd., Tokyo, Japan) that was used to move the transducer so as to place the target plate at the focal point of the transducer. A pulse-receive system (UT340, UTEX Scientific Instruments Inc., Mississauga, ON, Canada) was used to excite the transducer for ultrasound transmission and to receive the echo from the target plate. The received signal was digitized and recorded using an oscilloscope (DPO7054, Tektronics Inc., Beaverton, OR, USA). The echo samples were used to evaluate the spectral characteristics of the developed transducer in MATLAB (Mathworks Inc., Natick, MA, USA). Figure 4 is the measured pulse echo response and frequency spectrum of the developed transducer. The center frequency and −6 dB fractional bandwidth was 37.3 MHz and 62% (i.e., from 25.7 to 48.8 MHz), which is in good agreement with the simulation results shown in Figure 2.

**Figure 5:**
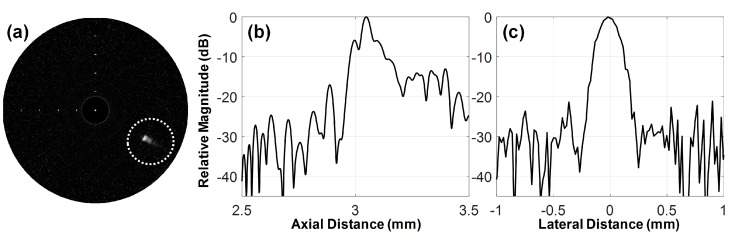
(**a**) Ultrasound B-mode image of a 25 μm wire acquired at a focal depth of 3 mm, (**b**) the axial and (**c**) lateral beam profiles measured from the wire image indicated by a white dotted circle.

The spatial resolution of the developed transducer was evaluated through a wire target imaging test. Instead of the target plate, a 25 μm wire was immersed into the container on the rotary stage (SGSP 160-YAW, SIGMAKOKI Co. Ltd., Tokyo, Japan). The wire was placed along the vertical direction and its location was adjusted so that ultrasound could meet the wire at the focal point of the transducer. The pulse-receive system was used to enable the developed transducer to transmit ultrasound and receive the echo from the wire. The digitization and recording of the echo signals were conducted using a Gage card (CS12502, Gage Applied Technologies Inc., Montreal, QC, Canada) in a personal computer. To acquire 1000 scanlines for one image, the rotary stage was rotated in 0.36°, which was controlled by a program written in LabView (National Instrument, Austin, TX, USA). Backend processing was performed in MATLAB to construct an ultrasound image [31]; logarithmic compression with a dynamic range of 30 dB was conducted for efficient visualization of the image as shown in Figure 5a. The −6 dB beam widths were measured from the axial and lateral beam profiles (see Figure 5b,c); those were 58 and 211 μm, respectively. Note that the axial beam profile shown in Figure 5b has a high level of the side lobes due to ultrasound reverberation from the wire.

### 3.2. Feasibility Study Using Custom Blood-Vessel-Mimicking Phantoms

**Figure 6:**
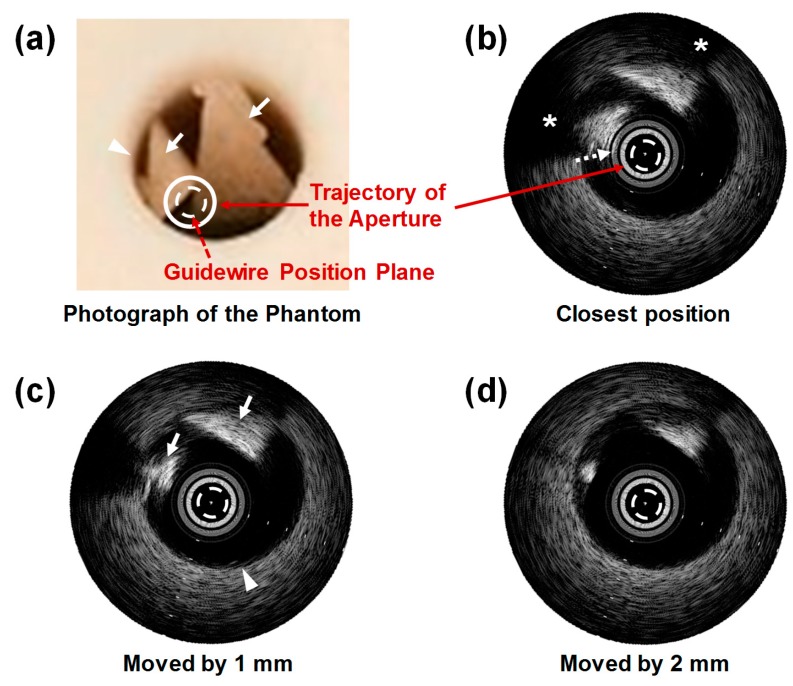
(**a**) Photograph of the custom blood-vessel-mimicking phantom and (**b**–**d**) FL–IVUS images inside the hole of the phantom (see the white solid circle in (**a**)). The white solid arrows in (**a**) indicate hyperechogenic regions (i.e., the calcification-mimicking parts), and the white arrow head is the boundary between the water and the tissue-mimicking phantom. The space between the white bars on the IVUS images indicates 1 mm in the slant height. The hole in (**a**) is 8 mm in diameter.

The ability of this FL–IVUS transducer to detect calcification in CTO lesions was ascertained using custom blood-vessel-mimicking phantoms constructed for this study. The phantom consisted of tissue- and calcification-mimicking parts. The tissue-mimicking region was made by mixing 8.0 g of agar powder (A9799, Sigma-Aldrich Co. Ltd., St. Louis, MO, USA) and 5.0 g of silicon dioxide powder (S5631, Sigma-Aldrich Co. Ltd., St. Louis, MO, USA). 250 mL of deionized water was heated to 60 °C on a hot plate. The detailed fabrication process of the tissue-mimicking phantom can be found in [32]. The mixture was added to the deionized water while vigorously stirring, which was cooled down and poured into a rectangular acrylic mold. After curing in a refrigerator for 4 h, a hole was created in the tissue-mimicking phantom. For the calcification-mimicking part, a mixture of 10.4 g of agar powder and 7.1 g of calcium carbonate (C4830, Sigma-Aldrich Co. Ltd., St. Louis, MO, USA) was added to 250 mL of deionized water. The detailed fabrication process can be found in [33]. Note that the calcium carbonate plays a role of calcification because this material is hyperechogenic. After solidifying, two small pieces of the calcification-mimicking phantom were attached to the hole surface in the tissue-mimicking phantom by using the 5 min Epoxy. In addition, one small piece or one large piece of the calcification-mimicking phantom was attached to each of the two different tissue-mimicking phantoms; the latter was especially constructed to mimic extensive calcification in CTO lesion. The hole was filled with deionized water. The photograph of the custom blood-vessel-mimicking phantom is shown in Figure 6a; two triangular-shaped calcification-mimicking phantoms are indicated by the two white solid arrows. FL–IVUS images of the phantom were acquired using the same system used for the wire-imaging test.

When a portion of the guidewire position plane was located above the calcified area as shown in Figure 6a, the bright image of the calcified area appeared in the vicinity of the aperture trajectory displayed in the FL–IVUS image, which is indicated by the white dotted arrow in Figure 6b. This happened when the FL–IVUS transducer was placed closest to the calcified area. The aperture trajectory was represented by the white solid circle in the FL–IVUS images (see Figure 6), and its radius is equal to the distance between the aperture surface and the vertex of the conical imaging plane, i.e., *d*_0_. Note that the gray circle next to the white circle in the FL–IVUS images stemmed from the low-frequency high-amplitude signals on which the echoes from the calcification-mimicking parts were loaded. These signals typically occur near the surface of a transducer due to the electrical noise and the ringing of electrical pulses. One possible solution to reduce the low-frequency high-amplitude signals is to use an electrical impedance matching circuit with a high-pass filter structure [34]. In general, no echoes are detected behind calcified lesions due to the high reflectance and attenuation of ultrasound in calcification. Therefore, the shadowing artifacts typically appear on the backside of calcified lesions in ultrasound images, whereas the calcification is very bright in ultrasound images [35]. The same phenomena were observed in the FL–IVUS images of the custom blood-vessel-mimicking phantom (the asterisks in Figure 6b). Although the echoes from the calcification-mimicking parts just below the guidewire position plane, indicated by the white dashed circle in the FL–IVUS images, could not be detected, it could be predicted from Figure 6b that the calcification-free area was likely to be located at 2 to 8 o’clock in the guidewire position plane. In reality, there was the calcified area below the guidewire position plane between 2 and 4 o’clock although this portion of the calcified area was located deeper than the rest (see Figure 6a). Therefore, the guidewire will meet the calcification if the surgeon selects the entry point of guidewire crossing in this direction. However, the FL–IVUS image not only provides information on the exact entry points that should be avoided, but also makes it possible to reduce the number of trials. For this reason, the FL–IVUS images facilitate an increase in the success rate of guidewire crossing. As the FL–IVUS transducer was moved backwards, the calcification images moved away from the aperture trajectory, but the position of the boundary between the tissue-mimicking phantom and the water, marked with the white arrow head in Figure 6c, did not change. This was so because the phantom had the cylindrical hole, and thus the distance between the transducer surface and the boundary was always the same regardless of the distance of the transducer from the calcified area if the FL–IVUS transducer was moved back straight.

**Figure 7:**
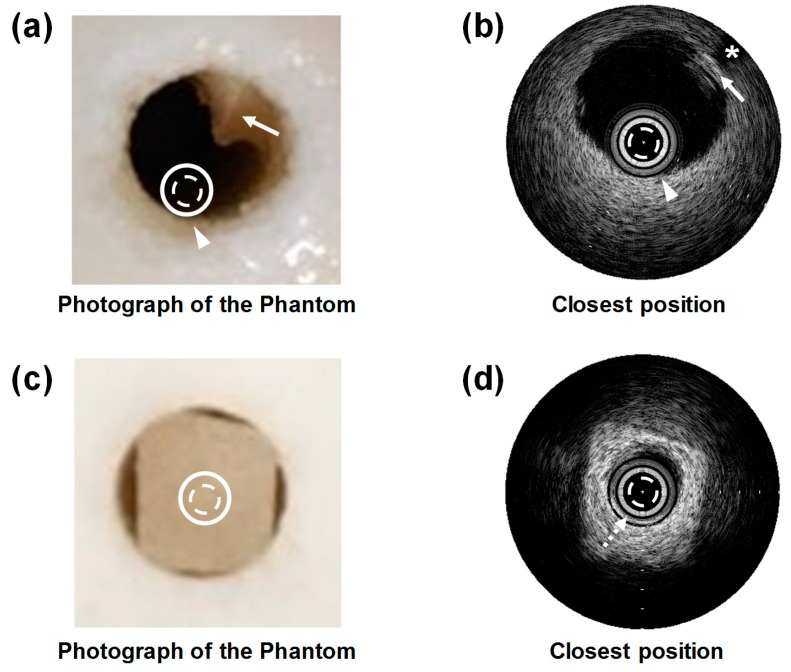
(**a**,**c**) Photographs of the custom blood-vessel-mimicking phantoms and (**b**,**d**) FL–IVUS images inside the hole of the each phantom. The white solid arrows in (**a**,**b**) indicate hyperechogenic regions (i.e., the calcification-mimicking parts), and the white arrow heads in (**a**,**b**) are the boundary between the water and the tissue-mimicking phantom. The space between the white bars on the IVUS images indicates 1 mm in the slant height. The holes in (**a**,**c**) are 5 mm in diameter.

When the FL–IVUS transducer was placed near the boundary (see Figure 7a), the boundary image appeared near the gray circle, but no acoustic shadows occurred as shown in Figure 7b. No shadowing artifact is the feature of the intima-lumen interface that appears on IVUS images; this is an indicator used to differentiate between the blood vessel wall and calcification. Therefore, the guidewire should not be advanced to the guidewire position plane between 4 and 8 o’clock. If this happens, a perforation of the blood vessel wall occurs. Additionally, Figure 7b showed that the calcified area was positioned away from the guidewire position plane and the safe entrance of guidewire crossing could be easily predicted. In the case of extensive calcification in CTO lesions, on the other hands, the bright image surrounded the gray circle and no water-phantom boundary (i.e., the intima-lumen interface) image appeared (see Figure 7d).

Since an imaging modality is not currently available to provide visual information inside CTO lesions, the surgeons repeatedly push and pull the guidewire to find a calcification-free area for guidewire crossing. Even when CTO lesions do not have calcification-free area, there is no way to provide this information to the surgeons. This is the reason why guidewire crossing requires a long operation time and a success rate is low. The developed FL–IVUS transducer can be used to solve the problem. When the FL–IVUS transducer reaches a CTO lesion, the surgeon drives a motor in an imaging system to rotate the transducer 360°. By doing so, one FL–IVUS image such as Figure 6b and Figure 7b can be constructed. Although conventional SL–IVUS imaging is performed by pulling and rotating a SL–IVUS transducer to obtain SL–IVUS images in real time, one FL–IVUS image suffices for guidewire crossing guidance. After identifying a calcification-free area in the FL–IVUS image, the transducer is rotated by a certain angle to position a guidewire at the area. Note that a FL–IVUS imaging system can be implemented to indicate the starting position of the rotation. Since a guidewire is always placed on the opposite side of the image scanline (i.e., a 180° angle difference as shown in Figure 1b), the rotation angle can be easily calculated to locate the guidewire at the calcification-free region. If the FL–IVUS image does not contain a calcification-free area, the surgeon can pull and push the transducer again to obtain another FL–IVUS image at a different location on the CTO lesion. However, the number of pulling and pushing operations can be considerably reduced due to the FL–IVUS image.

Since blood flow is not present in front of CTO lesions, it can be considered that there is no change in the transducer position after one rotation for image acquisition. Thus, a guidewire is expected to be placed at a desired position when the transducer is rotated by a certain angle calculated from a FL–IVUS image, although the guidewire cannot be visualized in the FL–IVUS image. The results of the phantom experiments showed the feasibility of using the developed FL–IVUS transducer to identify calcification areas inside CTO lesions, but in-vivo experiments should be conducted to evaluate the performance and clinical usefulness of the transducer. For this, CTO animal models as well as an imaging system should be developed, which is our future work.

## 4. Conclusions

In the paper, it was demonstrated that the 40-MHz FL–IVUS transducer consisting of a single element angled aperture and a guidewire passage can be used for visualization of calcification within CTO lesions. For this purpose, the aperture was spherically deformed to have a focal length of 3 mm in order to improve spatial resolution of FL–IVUS images, and the angle between the beam direction and the axis of rotation was designed to be 30° to effectively visualize calcification within a CTO lesion as well as the blood vessel wall. Although the pathway of guidewire crossing did not appear in the FL–IVUS images, the phantom experiment results demonstrated that the surgeons can predict from the FL–IVUS image whether calcification is in the pathway of guidewire crossing when a CTO lesion is close to the developed FL–IVUS transducer. In other words, the surgeons can know at least the entry points that should be avoided if the calcification sites are shown in the FL–IVUS images. Therefore, the FL–IVUS imaging with the developed transducer has the potential to help increase the success rate of guidewire crossing, compared to angiography that cannot provide information about internal CTO lesions.

## Figures and Tables

**Table 1:** Material properties and thicknesses of the piezoelectric material, the acoustic matching layers, and the backing block used for the proposed forward-looking intravascular ultrasound (FL-IUVS) transducer.

Parameters	PZT-5H	1st Matching	2nd Matching	Backing Layer
Longitudinal Velocity (m/s)	4700	1900	2350	1850
Density (g/cm^3^)	7.8	3.86	1.1	3.2
Acoustic Impedance (MRayl)	36.7	7.334	2.59	5.92
Clamped dielectric constant	1200	-	-	-
Thickness (μm)	59	13	16	414
